# The AI-Augmented Scientific Congress Ecosystem (AISCE): Reimagining Scientific Congresses in the Age of Artificial Intelligence

**DOI:** 10.7759/cureus.112331

**Published:** 2026-07-09

**Authors:** Joobin Khadamy

**Affiliations:** 1 Ophthalmology, Capio Ögon Stockholm Globen, Stockholm, SWE

**Keywords:** abstract review, artificial intelligence, conference management, digital twin, generative ai, knowledge transfer, medical congress, medical education, ophthalmology, scientific meetings

## Abstract

Scientific congresses remain central to medical education, innovation, networking, and clinical consensus, but they are increasingly challenged by rising abstract volumes, hybrid formats, reviewer fatigue, fragmented programming, repeated speaker networks, and limited post-congress knowledge transfer. This editorial proposes the AI-Augmented Scientific Congress Ecosystem, a human-supervised model in which artificial intelligence supports the congress lifecycle rather than replacing scientific committees, reviewers, moderators, or societies. Near-term applications include abstract triage, reviewer matching, duplicate and similarity screening, program scheduling, hall allocation, attendee guidance, multilingual access, live transcription, question clustering, retrieval-grounded literature support, and post-congress synthesis. More advanced future applications include live session AI-generated debate prompts, speaker discovery, future topic prediction, congress knowledge graphs, consensus-draft generation, and consent-based digital legacy archives for medical educators. A key paradox is that congresses often contain unpublished clinical experience, early data, surgical insights, and expert debate that may precede the indexed literature on which many AI systems depend. Therefore, AI should help structure emerging knowledge, not replace expert interpretation. Ophthalmology is a suitable model field because it is visual, quantitative, technology-driven, and already engaged with AI in imaging, diagnostics, surgical planning, simulation, and education. Responsible implementation requires source grounding, auditability, privacy protection, bias monitoring, consent, conflict-of-interest governance, and final human decision-making.

## Editorial

Scientific congresses are among the most important traditions in medicine. They are where new ideas are presented, where early data are tested in front of colleagues, where young researchers find visibility, and where clinical communities decide which innovations deserve attention. For many physicians, congresses are not only educational events but also places where careers, collaborations, and professional identities are shaped.

Yet the congress model has not changed as much as the science it presents. Programs are often prepared months in advance. Abstracts are reviewed under time pressure. Popular speakers are repeatedly invited because they are already known. Similar sessions may be placed in parallel halls, forcing attendees to choose between closely related topics. Valuable discussions may disappear once the session ends. Post-congress knowledge transfer is often limited to recorded lectures, PDF programs, and scattered personal notes.

**Figure 1:**
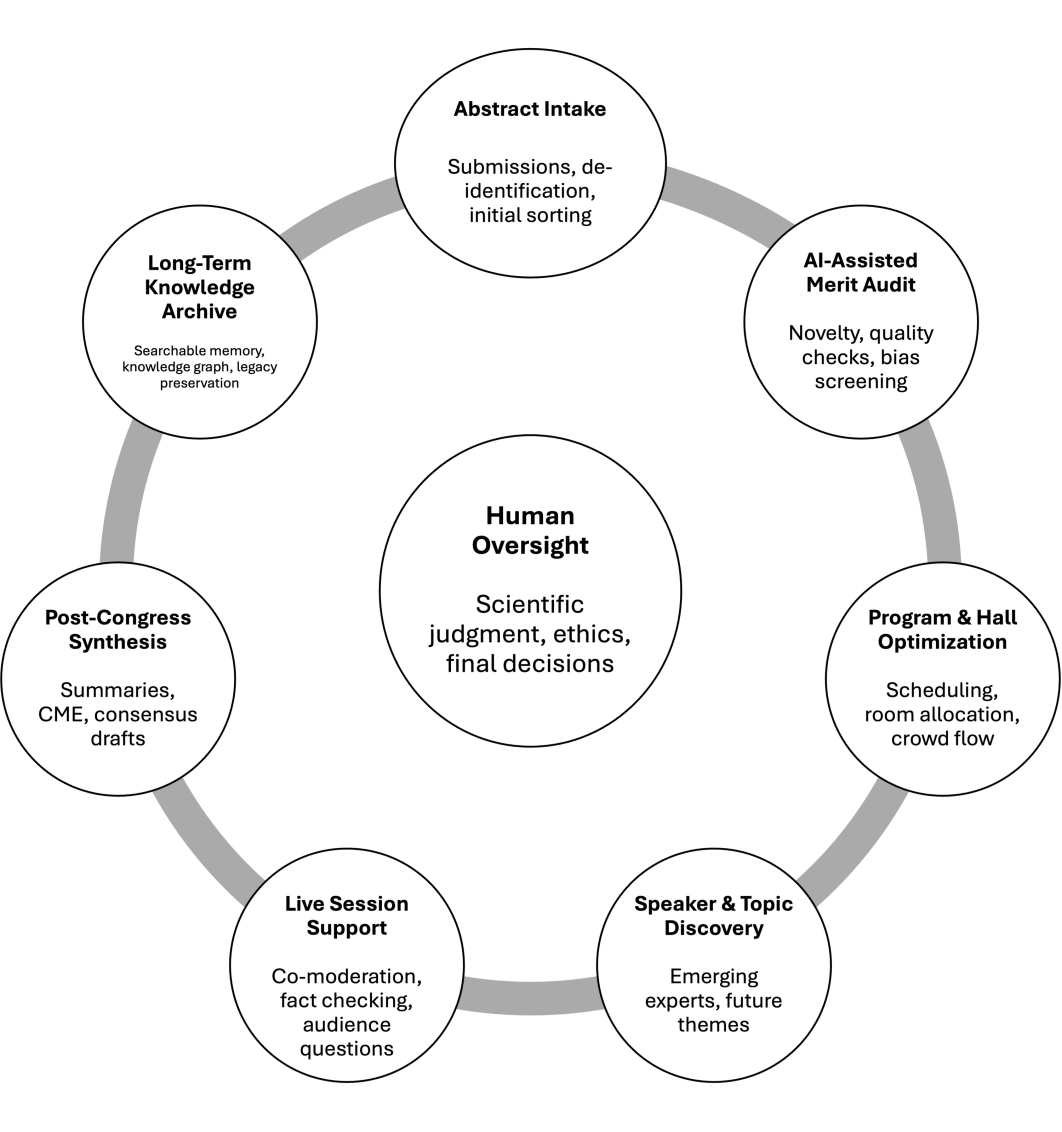
The AI-Augmented Scientific Congress Lifecycle The figure illustrates seven stages in the proposed AI-Augmented Scientific Congress Ecosystem, with human oversight placed at the center of the lifecycle. AI may support abstract intake, merit auditing, program and hall optimization, speaker and topic discovery, live session support, post-congress synthesis, and long-term knowledge archiving. AI functions as a co-pilot, not an autonomous authority. Image Credits: Joobin Khadamy; created manually in Microsoft PowerPoint (Microsoft Corporation, Redmond, WA, US)

Artificial intelligence (AI) may help us rethink this model. The goal should not be to replace congress committees, reviewers, moderators, or scientific societies. Medical congresses need human judgment, intellectual leadership, ethical responsibility, and clinical nuance. However, AI can become a powerful co-pilot across the congress lifecycle, supporting selection, organization, moderation, personalization, and memory [1]. The major AI-supported domains across this lifecycle are summarized in Figure 1, which places human oversight at the center of abstract intake, merit auditing, program optimization, speaker and topic discovery, live session support, post-congress synthesis, and long-term knowledge archiving.

The possible roles of AI across the congress lifecycle are broad and should be considered together with their potential benefits, risks, and safeguards. Table 1 summarizes these domains, from abstract review and scientific programming to live moderation, post-congress learning, and preservation of educational legacy.

**Table 1:** Potential applications, benefits, risks, and safeguards of artificial intelligence across the scientific congress lifecycle Examples are illustrative and include conference-management platforms, literature databases, bibliometric tools, retrieval systems, scheduling methods, digital-twin/venue-simulation tools, surgical-video analytics, and emerging commercial technologies. Mention of a named platform does not imply endorsement, regulatory validation, ophthalmology-specific validation, or routine use in medical congress workflows. Several applications remain proposed extrapolations and require prospective evaluation, human oversight, auditability, cybersecurity and privacy review, conflict-of-interest governance, consent where applicable, and final human decision-making. AI, artificial intelligence; CME, continuing medical education; CMT, Conference Management Toolkit; COI, conflict of interest; ORCID, Open Researcher and Contributor ID; RAG, retrieval-augmented generation; RDF, Resource Description Framework; SPARQL, SPARQL Protocol and RDF Query Language; TPMS, Toronto Paper Matching System

Congress Domain	AI-Supported Function	Example Tools or Approaches	Potential Benefit	Main Risk	Required Safeguard
Abstract Review	De-identification, topic clustering, novelty scoring, quality checks, duplicate detection [1,2]	Ex Ordo, Oxford Abstracts, Converia, X-CD, Fourwaves, Dryfta; blind/double-blind review workflows; iThenticate/Crossref Similarity Check; semantic clustering and duplicate-screening models	More consistent and merit-based abstract assessment	Algorithmic bias, overreliance on automated scoring	Human final decision, audit trail, bias monitoring
Reviewer Assignment	Reviewer matching, expertise mapping, conflict detection [2]	OpenReview paper matching, Microsoft CMT/TPMS, Converia reviewer assignment, ORCID/PubMed/OpenAlex/Semantic Scholar-linked expertise profiles, declared COI forms	Better reviewer fit and fewer conflicts of interest	Incomplete reviewer profiles or missed conflicts	Committee verification and transparent conflict policy
Scientific Program	Topic discovery, keynote suggestion, gap analysis, speaker shortlisting [1]	PubMed, Semantic Scholar, Elicit, Consensus, and scite for literature/gap analysis; SciVal, InCites, Dimensions, OpenAlex, Lens.org, and VOSviewer for bibliometric and expert-network mapping; ORCID-linked profiles for researcher identification and disambiguation	More relevant, innovative, and balanced program	Citation bias, speaker recycling in algorithmic form	Transparent criteria, diversity review, conflict disclosure
Scheduling	Day planning, parallel-session optimization, speaker availability management	Integer/constraint-programming conference-scheduling models [3], ConfTool Pro, Sessionboard AI Agenda Builder, X-CD/Fourwaves/Converia program modules, scientific-committee manual adjustment	Fewer conflicts between related sessions and better use of congress time	Over-optimization or loss of human educational priorities	Manual override and scientific committee approval
Hall and Venue Planning	Room allocation, attendance prediction, crowd-flow simulation, and hybrid attendance planning	Digital-twin models [4], OnePlan Venue Twin, VenueTwin, Iventis/Virtual Venue-type platforms, TruVenue/inControl simulation, crowd-flow, and room-allocation analytics	Better room matching, fewer bottlenecks, improved attendee movement	Poor input data, privacy concerns from movement analytics	Privacy-preserving analytics and venue-team review
Live Sessions	AI co-moderation, question clustering, evidence retrieval, participation balancing [1,5]	Slido AI question clustering, Pigeonhole Live moderation/filtering, live transcription, Zoom/Teams translated captions, audience-response systems, retrieval-grounded moderator dashboard	More focused discussion and stronger audience engagement	Inaccurate prompts or inappropriate AI intervention	Chair control, source verification, no autonomous authority
Real-Time Fact Checking	Literature alerts, claim verification, trial and guideline retrieval [5]	RAG systems connected to PubMed, ClinicalTrials.gov, society guidelines, NICE/WHO guidance, trial registries, and curated congress materials	More evidence-grounded discussion and reduced unsupported claims	False correction or misinterpretation of retrieved sources	Expert validation before public use
Attendee Support	Personalized agenda, networking recommendations, missed-session summaries, multilingual assistance	EventPilot/ATIV, Whova/Bizzabo-type congress apps, Grip/Brella AI matchmaking, multilingual captions	Better navigation, inclusion, and personalized learning	Privacy risk and excessive profiling	Opt-in design, data minimization, clear privacy policy
Education and Content Creation	Lecture summaries, captions, simulation cases, surgical video annotation, invitation drafting [1,6,7]	NotebookLM/source-grounded summarization, speech-to-text captioning, translation tools, multimodal LLMs, ZEISS Surgery Optimizer-type ophthalmic surgical-video platforms, SurgeryView.ai/SurgicalVideo.io-type surgical-video analytics, cataract phase-recognition/instrument-detection models, consented avatar/video tools for narration only	Lower administrative burden and richer educational material	Copyright issues, inaccurate educational content	Consent, copyright review, expert verification
Post-Congress Learning	Searchable summaries, CME material, quizzes, consensus drafts, personalized learning paths [5]	RAG knowledge bases, NotebookLM, PubMed alerts, Covidence/Rayyan/RevMan/GRADEpro-type evidence-synthesis and guideline-development tools, CME quiz generators	Longer congress impact and improved knowledge transfer	Oversimplification or outdated summaries	Source grounding, version control, correction mechanism
Knowledge Architecture	Congress knowledge graphs linking speakers, abstracts, papers, trials, topics, and controversies [5]	Neo4j, Ontotext GraphDB, RDF/SPARQL stores, OpenAlex API, Semantic Scholar Academic Graph API, semantic search, vector databases, curated congress metadata	Converts the congress archive into an active map of knowledge	Misclassification or incomplete linkage	Curated metadata and periodic expert review
Legacy Preservation	Digital educator archives, teaching-style preservation, educational avatars based on approved material	Panopto/Kaltura institutional lecture repositories, consent-based voice/video archives, Azure Personal/Professional Voice, ElevenLabs-type voice models, Synthesia/HeyGen-type avatar platforms, retrieval-restricted educator archives	Preservation of tacit knowledge, teaching style, and reasoning patterns	Misuse of likeness or deceptive imitation	Explicit consent, clear labeling, limited educational use

The applications in Table 1 should be interpreted along a spectrum of maturity. Some functions, such as abstract-management workflows, reviewer assignment, similarity screening, literature retrieval, transcription, translation, audience-response tools, and post-congress summarization, are already close to routine implementation. Other applications, including AI debate opponents, autonomous consensus-draft generation, advanced congress knowledge graphs, digital-twin venue simulation, and digital educator archives, remain more forward-looking and should be considered proposed directions for development and evaluation rather than established medical-congress practice.

One of the first areas where AI can contribute is abstract selection. Traditional abstract review depends on human expertise, but it is also vulnerable to inconsistency, fatigue, institutional bias, language bias, geographic bias, and personal familiarity [2]. AI could assist by classifying abstracts by topic, identifying study design, detecting missing methodological details, flagging unclear outcomes, recognizing duplicate or overlapping submissions, and comparing claims of novelty with recent literature [1,2]. This would not mean that AI accepts or rejects abstracts. Rather, it could function as a scientific merit auditor, helping reviewers focus on methodological quality, originality, clinical relevance, and reproducibility.

This may be especially useful in large specialty meetings. In ophthalmology, for example, hundreds of abstracts may cover cataract surgery, retina, glaucoma, cornea, uveitis, refractive surgery, imaging, artificial intelligence, and surgical education. AI could help identify whether an abstract on anterior segment optical coherence tomography truly adds new information, whether a study on intraocular lens calculation reports clinically meaningful refractive outcomes, or whether a retinal imaging paper is genuinely innovative rather than another minor variation of previous work. In this way, AI may help congresses move away from reputation-based visibility and toward more merit-based selection.

AI can also help with program design. Scheduling a congress is an optimization problem: organizers must balance topic overlap, speaker availability, hall size, expected attendance, wet labs, industry sessions, poster sessions, audiovisual needs, and attendee movement between venues. AI could suggest program structures that reduce unnecessary conflict between related sessions. For instance, a session on AI in retinal imaging should not be placed at the same time as a session on AI in glaucoma progression if both attract similar audiences. A high-demand surgical video symposium should not be placed in a small hall while a narrow session occupies the main auditorium.

Digital twin technology could extend this idea further. A digital twin is a data-driven virtual representation of a physical entity, environment, or process that can be synchronized with real-world data and used for simulation, forecasting, optimization, or decision support [4]. Applied to a congress venue, a digital twin could simulate hall capacity, crowd flow, poster traffic, exhibition density, and movement between lecture rooms. This would allow organizers to test different layouts before the meeting begins. For large medical congresses with wet labs, imaging demonstrations, industry exhibitions, and parallel subspecialty programs, such planning could improve both educational quality and attendee comfort.

Speaker selection is another area where AI could be useful, but also where caution is needed. Invited lectures often depend on reputation, seniority, and professional networks. These factors matter, but they can also lead to speaker recycling and missed emerging voices. AI could scan publications, citation patterns, trial leadership, educational output, patents, guidelines, and conference archives to suggest both established and rising experts. It could also help committees identify underrepresented regions, younger investigators, and cross-disciplinary contributors. The final decision should remain human, but AI can widen the field of view.

AI may also help predict future congress topics. Instead of only responding to what is already popular, societies could use Agentic AI to monitor PubMed, preprints, clinical trial registries, grant databases, patents, regulatory approvals, and industry pipelines. In ophthalmology, such systems might detect early growth in lens capsule bioengineering, home tonometry, intraoperative OCT, robotic microsurgery, multimodal retinal prediction, AI-guided refractive planning, or anterior segment OCT biomarkers in uveitis. A congress should not only reflect the field as it was last year; it should help prepare the field for where it is going next.

This also reveals an important paradox. Many current AI systems rely on existing text, whether through training data, indexed literature, or retrieval from available databases [1,5]. Their knowledge may therefore lag behind the newest unpublished findings, early surgical experience, preliminary trial results, failed techniques, expert disagreements, and informal clinical insights that often appear first at congresses. This is particularly relevant because results initially presented as conference abstracts are not always subsequently published as full articles, meaning that part of the knowledge exchanged at congresses may remain outside the indexed peer-reviewed literature [8]. In this sense, congresses may remain ahead of AI because they capture knowledge before it becomes a paper, guideline, or searchable database entry. The most useful future systems will not simply summarize published literature; they will help transform the congress itself into a structured source of emerging knowledge.

During the congress itself, AI could support live sessions. The most realistic model is not an autonomous AI moderator, but an AI-assisted human moderator. The system could listen to a lecture, identify the main claims, summarize audience questions, detect unanswered issues, and suggest follow-up questions to the chair. It could also compare a speaker’s statement with recent literature and quietly alert the moderator when a claim of novelty or superiority may require qualification [1,5].

A more advanced version would be the AI debate opponent. This does not mean creating an aggressive or theatrical machine voice. It means giving the moderator scientifically useful, source-grounded prompts generated through retrieval-augmented methods, while preserving human control over interpretation [1,5]. After a presentation, the AI might suggest: “The conclusion assumes causality, but the data appear associative”; “The study did not stratify outcomes by baseline disease severity”; or “Recent studies have reported conflicting results.” Used carefully, this could make discussions more rigorous and less dependent on whether someone in the room happens to remember the relevant literature.

Real-time fact-checking is attractive but must be handled with humility. AI can retrieve relevant studies, clinical trials, or guidelines, but it can also misinterpret sources or generate confident errors [1,5]. Therefore, real-time literature support should be presented as possible relevant context, not as definitive correction. The human moderator must decide whether and how to use it.

AI could also improve the attendee experience. Large congresses are difficult to navigate, especially for trainees, first-time participants, and international visitors. A personal AI congress companion could recommend sessions, posters, wet labs, and networking opportunities based on an attendee’s specialty, interests, prior publications, bookmarked sessions, and learning goals. It could summarize missed lectures, translate key points, generate reading lists, and suggest colleagues with shared interests. This would make the congress more personalized and inclusive.

After the congress, AI may be even more valuable. Many meetings generate large amounts of knowledge that are poorly preserved. AI could transform lectures, discussions, posters, and audience questions into searchable summaries, educational modules, podcasts, journal club material, and continuing medical education content. It could also build a congress knowledge graph, linking speakers, abstracts, papers, trials, topics, controversies, and future research questions. Instead of searching through isolated recordings, attendees could ask: “What were the main controversies in glaucoma surgery this year?” or “Which sessions discussed AI in diabetic retinopathy screening?” The congress archive would become an active map of knowledge rather than a passive storage system. By converting live congress material into curated, searchable, and version-controlled knowledge, AI could help narrow the gap between rapidly emerging expert knowledge and the slower pace of formal publication.

**Figure 2:**
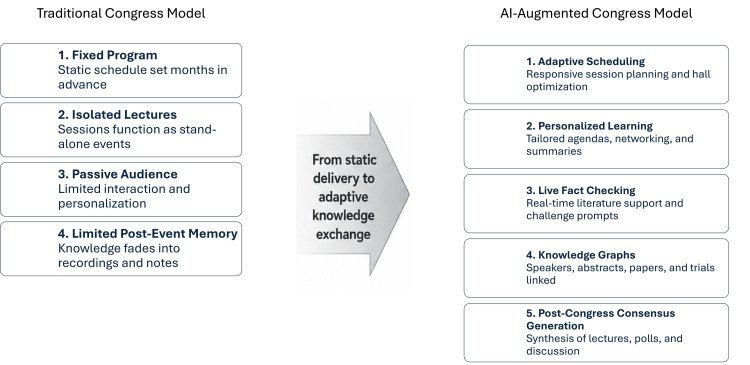
From static congress to living knowledge system The figure contrasts a traditional congress model, characterized by fixed scheduling, isolated lectures, passive participation, and limited post-event memory, with an AI-augmented model based on adaptive scheduling, personalized learning, live fact checking, knowledge graphs, and post-congress consensus generation. AI enhances scientific exchange while human experts remain responsible for judgment and interpretation. Image Credits: Joobin Khadamy; created manually in Microsoft PowerPoint (Microsoft Corporation, Redmond, WA, US)

AI may also help with consensus development. At the end of a meeting, it could synthesize lectures, panel discussions, audience polls, abstracts, and current literature into a first draft of a consensus statement or meeting report. Experts would then revise, debate, and approve the final text. This could shorten the time between scientific discussion and practical guidance, while preserving expert accountability. This shift is illustrated in Figure 2, which contrasts the traditional congress model of fixed programs, isolated lectures, passive audiences, and limited post-event memory with an AI-augmented model based on adaptive scheduling, personalized learning, live fact checking, knowledge graphs, and post-congress consensus generation.

**Figure 3:**
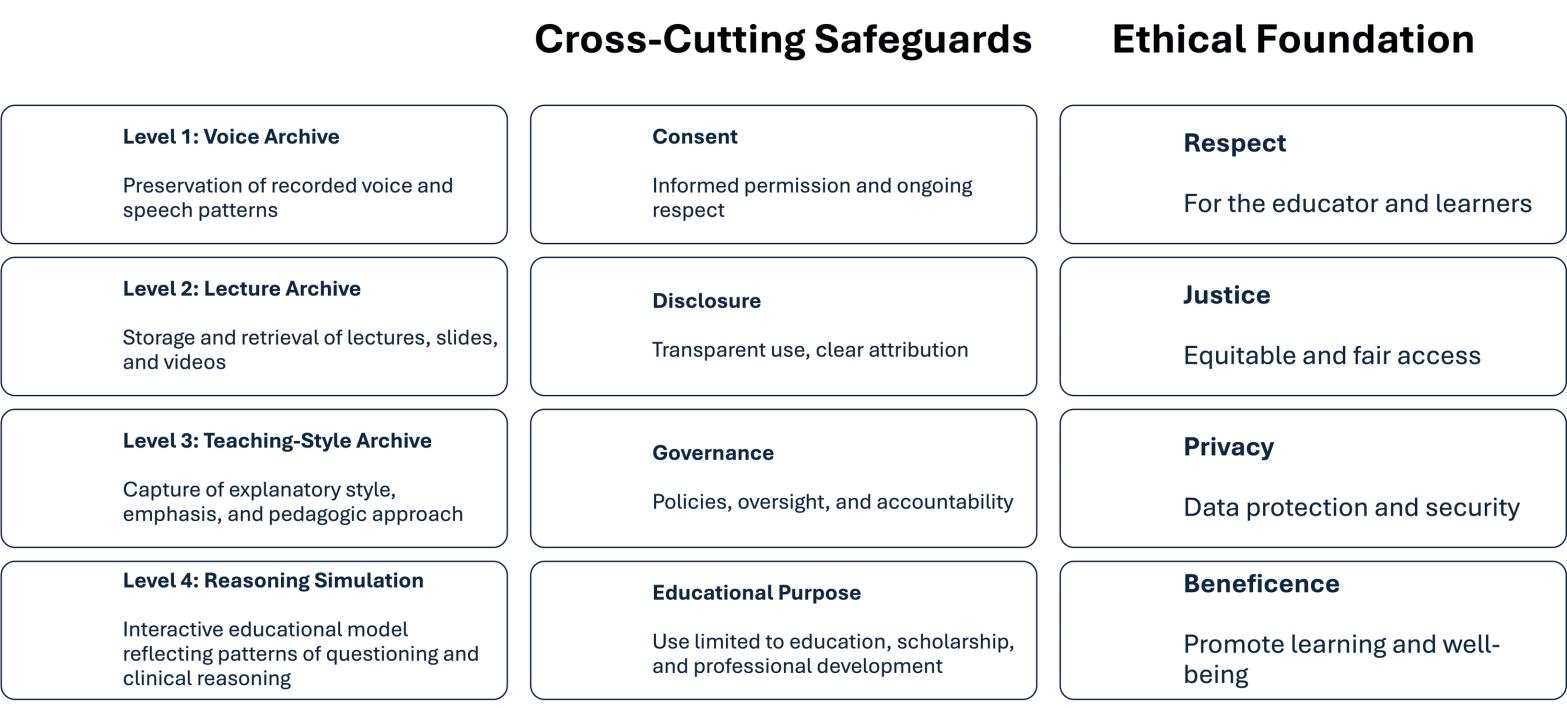
Digital legacy framework for medical educators The figure presents a tiered framework for preserving the educational legacy of medical educators, progressing from voice archiving to lecture archiving, teaching-style preservation, and reasoning simulation. The model emphasizes consent, disclosure, governance, educational purpose, respect, justice, privacy, and beneficence as safeguards across all levels. Digital preservation should support education, not deceptive imitation. Image Credits: Joobin Khadamy; created manually in Microsoft PowerPoint (Microsoft Corporation, Redmond, WA, US)

One of the most original and sensitive possibilities is the preservation of educational legacy. Medicine is not transmitted only through articles and slides; it is also transmitted through voice, operative judgment, teaching style, clinical intuition, and the questions great mentors ask. When influential clinicians and educators retire or pass away, much of this tacit knowledge disappears. With explicit consent and clear disclosure, AI could help create digital educational archives that preserve not only lectures, but also teaching style and reasoning patterns. A trainee might one day learn how a master cataract surgeon approached a difficult case, how a glaucoma expert questioned a visual field progression study, or how a retina specialist interpreted complex optical coherence tomography (OCT) findings. A possible framework for this ethical preservation of educational legacy is shown in Figure 3. The model progresses from basic voice archiving to lecture archiving, teaching-style preservation, and reasoning simulation, while requiring consent, disclosure, governance, and a clearly defined educational purpose at every level.

This idea must be governed carefully. The aim should not be deceptive imitation or commercial use of a person’s likeness. It should be transparent preservation for education. Any digital representation of a real expert should require consent, labeling, institutional oversight, and protection against misuse. Properly handled, however, this could allow future generations to learn not only what great physicians knew, but how they thought.

Ophthalmology is an ideal field for piloting these ideas. It is visual, quantitative, surgical, imaging-rich, and already closely connected to AI. The specialty has seen major AI work in diabetic retinopathy screening, retinal imaging, glaucoma assessment, intraocular lens calculations, surgical simulation, and workflow support [6]. Ophthalmology congresses also contain multiple educational formats: live surgery discussions, video symposia, imaging workshops, wet labs, poster sessions, technology demonstrations, and subspecialty debates. These features make ophthalmology a natural testing ground for AI-augmented congress design.

Practical implementation would also require attention to feasibility. AI-augmented congress systems may entail substantial costs, procurement barriers, cybersecurity requirements, data governance reviews, staff training, interoperability with existing congress platforms, and clear rules on data ownership. Societies would need to decide whether abstracts, slides, recordings, audience questions, chat logs, and attendee-behavior data can be processed by AI systems, where those data are stored, who can access them, and how long they are retained. These barriers are especially important for unpublished abstracts, preliminary trial data, industry-sponsored sessions, and recordings of identifiable speakers or patients.

The risks are real. AI can hallucinate, amplify bias, expose confidential data, overvalue citation metrics, favor English-language literature, or make subjective decisions appear objective [1,5,9]. Unpublished abstracts and slides must not be uploaded into insecure public tools without permission. AI-assisted decisions should be auditable, and societies should monitor whether AI recommendations create unfair patterns across geography, gender, language, institution, subspecialty, or commercial affiliation. Synthetic voices and avatars require especially strict consent and transparency [9].

The best future congress will not be an automated congress. It will be a human scientific community strengthened by carefully designed AI systems. AI can help identify better science, reduce administrative burden, improve scheduling, support live discussion, personalize learning, preserve knowledge, and extend the meeting's lifespan beyond a few days. But the responsibility for meaning, judgment, ethics, and clinical translation must remain with humans.

The question is therefore not whether AI should be present at medical congresses. It already will. The more important question is whether scientific societies will use it passively and inconsistently, or deliberately and ethically. If adopted with transparency, oversight, and humility, AI could help congresses become more merit-based, inclusive, interactive, evidence-grounded, and enduring. In ophthalmology and beyond, the congress of the future should not simply display knowledge. It should organize it, challenge it, preserve it, and help it reach the people who need it most.
